# Metabolite Storage in *Theobroma cacao* L. Seed: Cyto-Histological and Phytochemical Analyses

**DOI:** 10.3389/fpls.2019.01599

**Published:** 2019-12-05

**Authors:** Martina Cerri, Lara Reale, Claudia Zadra

**Affiliations:** ^1^Department of Agricultural, Food and Environmental Sciences, University of Perugia, Perugia, Italy; ^2^Department of Pharmaceutical Sciences, University of Perugia, Perugia, Italy

**Keywords:** antioxidant, cotyledons, histology, lipids, polyphenols, teguments

## Abstract

Cocoa (*Theobroma cacao* L.), an economically important tropical-fruit crop as source of chocolate, has recently gained a considerable attention; its seeds contain a large amount of different bioactive compounds that have attracted interest because may be beneficial to humans by improving cardiovascular health, by cancer chemo-preventive effects and also through neuroprotective activities. The morphological and anatomical characteristics of cocoa seeds are closely related to the aroma and to the nutritional properties. This study aimed to provide more information about the storage of some metabolites in the various components of cocoa seed by microscopical and phytochemical analyses. Polyphenols, sterols, tocopherols and fatty acids were detected in different portions of the seeds (teguments, cotyledons, embryo axis and pulp). Quali and quantitative differences were observed and a characteristic polyphenol pattern was detected in the different portions of the seed; cytological analysis demonstrated the presence of these compounds in big vacuolated polyphenolic cells. Among the analyzed fatty acids, the stearic and oleic acids were the most abundant in all the seed components (teguments, cotyledons and embryo axis). Fatty acids, usually found in the form of esters, thioesters and amides, represent one of the storage substances of cocoa seed probably localized in lipid globules, which in our observations occupied almost the entire volume of small isodiametric cells of cotyledon mesophyll. In the cocoa seeds we observed also a different distribution of sterols: β-sitosterol and Δ5-avenasterol were the most abundant, above all in the embryo axis; stigmasterol and campesterol were less present in embryo axis and more abundant in teguments; campestanol level was again higher in teguments but lower in cotyledons. The specific localization of different kind of sterols was probably related to a peculiar function. Our experiments demonstrated that all seed components contribute to the metabolites storage, but with interesting differences in the localization and amount of each metabolite.

## Introduction

Cocoa (*Theobroma cacao* L.) belongs to the family Sterculiaceae. It is an economically important tropical-fruit crop, mainly known as the source of chocolate and was introduced to Europe during the 16th century.

The fruit of cocoa tree is a pod, or cherelle, which shape and colour can differ among morphogenetic groups. Each pod holds 20 to 60 seeds, or beans, embedded in a white pulp, and is constituted by a thick epicarp, of variable hardness and with a pigmented epidermis, a thin and hard mesocarp, more or less woody, and finally the endocarp (Bertazzo et al., 2013). The endocarp is composed by big tubular cells with large intercellular spaces which, in the ripe seeds, collapse and form the so-called pulp. Pulp is rich in water and nutrients, contains 10–15% of sugar, is characterized by a low pH (3.6–4.0) and plays an important role during the seed fermentation, contributing to the flavour development (Hui et al., 2006).

The potential health implications of biologically active substances present in cocoa seeds are well documented. They are rich in natural antioxidants, such as polyphenols and tocopherols. Thanks to this antioxidant property, many of these compounds, especially flavonoids, exhibit also a wide range of pharmacologic effects, including antibacterial, antiviral, anti-inflammatory, antiallergic, antithrombotic and vasodilatory actions (Cook and Samman, 1996; Batista et al., 2016). The high levels of flavanols are responsible for the bitterness of cocoa that represent a fundamental aspect of the organoleptic and palatability characteristics of chocolate, and contribute to cocoa health benefits (Jalil and Ismail, 2008).

Epicatechins (that are classified as flavan-3-ols, based on their structure) are the most abundant cocoa phenolic components; they mainly include monomeric (−) epicatechin and (+) catechin (as well as oligomeric and polymeric proanthocyanidin flavanols), gallocatechin and epigallocatechin (Ortega et al., 2008).

Epicatechin represents approximately 35% of polyphenol content of unfermented Forastero cocoa beans (Othman et al., 2010). The antioxidant properties are also related to the presence of tocopherols and tocotrienols, which reduce oxidative stress and delay the progress of a variety of degenerative disorders, such as cardiovascular diseases and cancer. In addition, they have been shown to regulate cellular signalling, cell proliferation and gene expression (Sen et al., 2007). The total tocopherol content in cocoa beans is reported to be in a range of 100–300 mg/Kg fat (Carpenter et al., 1994; Shukla et al., 2005), values that are similar to those generally observed for wheat germ oil (Beliz and Grosch, 1999). The predominant isomer in cocoa bean is the gamma-tocopherol and a different distribution of the four isomers in seed parts of *Theobroma subincanum* were described by Bruni et al., 2002.

Precursors of tocopherols and polyphenols are produced from the plant primary biosynthetic shikimate and acetate pathways. The main products of these pathways are the aromatic amino acids phenylalanine (Phe), tyrosine (Tyr) and tryptophan (Trp) and acetyl-coenzyme A (CoA), respectively. They represent the starting molecules for the biosynthesis of a wide range of secondary metabolites (Tzin et al., 2012).

Other interesting classes of cocoa bioactive constituents are represented by phytosterols (collective terms comprising saturated sterols, also known as stanols) and fatty acids. Phytosterols are typical plant lipids which have a structural similarity with cholesterol and inhibit its intestinal absorption, contributing to a lower total plasma cholesterol and low-density lipoproteins levels. In cocoa seeds, the content of plant sterols is 2–3 mg/g fat, with an abundance of β-sitosterol and stigmasterol (Staphylakis and Gegiou, 1985) . Fatty acids are organized as triacylglycerol (TAG), the majority of these TAG’s being 2-oleyl glycerides (O) of palmitic (P) and stearic (S) acids (POP, POS, SOS) (Simoneau et al., 1999; Segall et al., 2005). This TAG structure directly affects the way chocolate behaves in the manufacturing process and the characteristics of the final product (texture, viscosity, melting behaviour, flavour and taste) (Afoakwa, 2010). The fatty acids content depends on the variety and region of cultivation of cocoa beans (Bruni et al., 2002; Torres-Moreno et al., 2015).

The biosynthesis of fatty acids requires as precursor the acetyl-CoA, that also represents the starting point of the mevalonate pathway from which arise the secondary metabolites, sterols/phythosterols.

The presence of above-mentioned compounds (as polyphenols, phytosterol and fatty acids) is not the only trait influencing the nutritional characteristics and aroma of cocoa beans, their morphological and anatomical traits, as the permeability of the seed coat, are also very important aspects. Andersson et al. (2006) suggested that water and solutes flow through seed coat during the fermentation process was strongly related to the flavour quality. The acetic acid produced during the fermentation moves thought the seed coat to contribute to formation of flavour precursors in the cotyledons. The uptake of acetic acid is also influenced by the fruit pulp and the inner pulp epidermis. The cyto-histological characteristics of cotyledons parenchyma are also important; the size of cells can influence the diffusion of acetic acid into the seed, because cells with higher volume can provide larger spaces for chemical reactions (Biehl, 1973; Biehl et al., 1977). After a comparison between different clones and varieties of cocoa, Elwers et al. (2010) suggested that ‘the larger polyphenol and storage cells of Criollo seeds may contribute to the unique quality of this fine flavour cocoa’. Considering the important role of histo-anatomical traits, microscopy studies can be very useful to understand how metabolic processes are compartmentalized in plant tissues (Walker et al., 2001). The aim of this study is to better investigate the cyto-histological characteristics of cocoa seeds related to their composition in secondary metabolites, such as polyphenol and tocopherols. To our knowledge, there are in literature many data about the histological characterization of cocoa seeds (Martini et al., 2008; Elwers et al., 2010), as well as biochemical studies about its phenolics content and antioxidant capacity (Aprotosoaie et al., 2016; Endraiyani et al., 2016; Wang et al., 2016), but a specific localization of the phytochemicals in the different structures of seed and fruit has not been carried out yet. Metabolite localization was focused until now in the cotyledon structure, but, as previous described, also other structure of seed and fruit are important for the flavour development and nutritional characteristics. Knowledge about chemical composition and metabolite storage in the fruit wall of seed tegument can be also useful for the possible use of residual biomass originated during cocoa processing and for the presence of anticancer agents in these non-edible portions of cocoa (Zainal et al., 2014). Our research supplies a more complete picture of metabolites localization in all portions of cocoa seed and fruit.

## Materials and Methods

### Plant Material

Samples were collected from fresh fruits of cocoa, harvested at maturity, coming from the municipality of Tucupita, Delta Amacuro state, Venezuela, and kindly donated to our University. The cocoa plantations of the Delta are established essentially in the tropical humid forest, in fertile alluvial soils with problems of poor drainage and in some units of tropical dry forest, with humid edaphic associations (Reyes and Capriles De Reyes, 2000; Gómez and Azócar 2002; Ministerio del Poder Popular para la Ciencia y Tecnología (MPPCT), 2006), in this area in general the soils have drainage problems and are flooded at certain times of the year (Corporación Venezolana de Guayana (CVG), 2006).

Seeds were separated from the rest of the fruit and their different components were progressively isolated. When gently removed from the fruit, seeds conserve mucilage, a portion of the modified endocarp; seeds with mucilage represent the first sample of this study (SM). In other seeds, mucilage was removed and seeds without mucilage were considered (S); the last samples were represented by the only teguments (T), embryo axis (EA) and cotyledons (C) isolated from seeds. All the samples were pulverized with liquid nitrogen and silicon dioxide in a mortar.

### Cyto-Histological Observations

Cocoa seeds were collected from a fresh fruit of cocoa; portions of fruit pericarp, seed T, C and embryo were fixed in 3% (w/v) glutaraldehyde in 0.075 M cacodylate buffer, pH 7.2, for 24 h. The samples were then washed three times for 7 min in 0.075 M cacodylate buffer, pH 7.2, post-fixed in 1% (w/v) OsO_4_ in the same buffer for 1 h, dehydrated in increasing concentrations of ethanol and finally embedded in epoxy resin (Epon, 2-dodecenylsuccinic anhydride and methylnadic anhydride mixture) (Reale et al., 2017, with modifications). Semi-thin sections (1–2 µm), obtained with an ultramicrotome (OmU2, Reichert, Heidelberg) after staining were observed under a light microscope (BX53; Olympus, Tokyo, Japan).

#### Periodic Acid Schiff’s Reaction

Semi-thin sections were treated with 0.5% periodic acid for 30 min at 40°C, washed with tap and demineralized water and covered with Schiff’s reagent for 15 min (O’Brien and McCully, 1981). Sections were then washed rapidly with tap water, two times for 3 min with SO_2_ water and two times for 10 min with demineralized water. Sections were also counter-stained with 1% (w/v) amido black in 7% acetic acid for protein. The presence of proteins was indicated by a blue colour, whilst starch grains appeared magenta.

#### Toluidine Blue Staining

Semi-thin sections were covered with 0.5% (w/v) toluidine blue in 2% NaHCO_3_ buffer. Toluidine blue has a high affinity for acidic tissue components and stains nucleic acids blue and polysaccharides purple (Feder and O’Brien, 1968).

#### Toluidine Blue O Staining

Semi-thin sections were stained with 0.5% (w/v) toluidine blue O in 0.1 M phosphate buffer, pH 7.2. The metachromatic stain develops a green-blue colour when associated with polyphenolic compounds, whilst turns pink with pectic substances and purple with nucleic acids and protein (Feder and O’Brien, 1968).

#### Vanillin Staining

Semi-thin sections were deresinated, stained for 15’ with vanillin (10% w/v) in ethanol mixed with 12 M HCl (2:1, v/v) (Earp et al., 2004), and mounted in glycerin. Vanillin turns up red upon binding to flavan-3,4-diols and flavan-4-ols (catechins), which are present either as monomers or as terminal subunits of proanthocyanidins.

### Chemicals

All the chemicals were of analytical grade and, unless otherwise specified, were purchased from Merck (Darmstadt, Germany).

### Chemical Analyses

For chemical analysis the considered samples (SM, S, T, C, EA) were collected from different seeds, pooled and analyzed in triplicates.

#### Analysis of Total Polyphenolic Compounds Content

The total phenolic compounds were extracted from defatted cocoa samples (SM, S, T, C, EA) (through exhaustive extraction with *n*-hexane) with a mixture of acetone and water 80:20 (v/v) in an ultrasonic bath for 15 min. After centrifugation the supernatant was filtered, and this procedure was repeated twice. The supernatants were combined, the acetone was evaporated under vacuum and the residue was analyzed for the total phenolic contents using Folin-Ciocalteu’s procedure (Singleton and Rossi, 1965). The total phenolic contents were calculated as a gallic acid equivalent (GAE) from a calibration curve of GA standard solutions and expressed as mg of GAE/g of sample.

#### Antioxidant Activity of Cocoa Polyphenol Extracts

The extracts were analyzed for their total antioxidant activity using the 2,2’-azino-bis(3-ethylbenzothiazoline-6-sulfonic acid (ABTS) method (Miliauskas et al., 2004). The ABTS^+^ radical was generated by oxidation of ABTS with potassium persulfate, then 1 ml of extract was added to the radical solution and the absorbance measured at 734 nm. Standard Trolox solutions were evaluated against the radical in order to obtain the calibration curve. The results of the antioxidant activity of cocoa extracts are expressed in terms of Trolox equivalent antioxidant capacity (TEAC) as mMol Trolox/g of sample.

#### High-Performance Liquid Chromatography-Analysis of the Extracts for Polyphenols and Tocopherols Identification

The samples of the previous aqueous extract derived from T, C and seed (with and without mucilage, SM and S) were analyzed by high-performance liquid chromatography (HPLC) for the identification of the polyphenolic compounds. Analyses were performed on a Perkin-Elmer PE 200 system (autosampler, binary pump and UV-Vis detector) equipped with an Inertsil 5 ODS-3 (250 mm x 4.6 mm i.d. x 5 µm, Varian) at a flow rate of 1.5 ml min^−1^; the injection volume was 20 µl, and detection was made in a spectrum from 280 to 530 nm for the different classes of compounds. The mobile phase consisted of (A) 1% acetic acid in water and (B) acetonitrile (80): water 1% acetic acid (20). The program was as follows: isocratic with solvent (B) for 20 min, linear gradient to 100% (A) for 15 min, 15 min linear gradient to solvent (B). The compounds were identified by comparing the retention times and areas with those of appropriate standards used for the calibration curve.

For the HPLC-analysis of the tocopherols in the T and C, these vegetable parts were suspended in 5 ml of methanol and sonicated (Branson 2800, Danbury, USA) for 20 min, the mixture was then filtered, rinsed with the solvent and evaporated under vacuum. The methanolic fractions were processed by a second extraction with 5 ml of n-hexane and dried under vacuum. HPLC analyses were performed on a Perkin-Elmer PE 200 system (autosampler, binary pump and UV-Vis detector) equipped with a LiChrosorb Si60 (250 mm x 4 mm i.d. x 5 µm column Phenomenex), the mobile phase was 0.05% isopropanol/hexane at a flow rate of 1 ml/min, the wavelength was 295 nm and the injection volume 20 µl. Tocopherols peaks from samples were identified by comparing the retention times with those of authentic reference compounds (tocopherols mix pure standards, Sigma-Aldrich). The compounds were quantified by an external standard method, using a calibration curve. For each extract, quali-quantitative analyses were performed in triplicates.

The lack of a sufficient amount of sample has not allowed to perform these analyses in the embryo.

#### Analysis of Fatty Acids

In the samples T, C, EA the lipid fraction (from hexane extraction) was subsequently derivatized into fatty acid methyl esters (FAMEs) by mild alkaline methanolysis. FAMEs were extracted twice with 2 ml aliquots of hexane:chloroform (4:1 v/v) and the pooled aliquots were dried under N_2_ at room temperature and redissolved in hexane-containing methyl nonadecanoate (C19:0) as internal standard. FAMEs were analyzed by gas chromatography-flame ionization detector (GC-FID) detector (Trace 2000, Thermo-Fisher) equipped with a 100% dimethyl-polysiloxane non-polar column (50 m length, 0.25 mm i.d. and 0.25 mm film thickness, Agilent J&W) and a split/splitless injector (1/10 ratio). The temperatures of the injector and detector were 220 and 250°C, respectively. The oven was temperature-programmed from 60°C (5 min) to 300°C (5 min) at a rate of 25°C/min. The injection volume was 1 µl. Preliminary peak identification was carried out by comparison of retention times with known standards. Relative amounts of given fatty acids were calculated from their respective chromatographic peak areas and the relative percentage of each fatty acid was related to the total peak areas of both saturated and unsaturated fatty acids.

#### Analysis of Phytosterols

In the samples T, C, EA the hexane extract was saponified with 2 M methanolic potassium hydroxide for 1 h at 70°C in ultrasonic bath, then was added 2 ml H_2_O and 3 ml hexane, vortexed and the two phases separated. Sterols were extracted from aqueous phase for three times. The organic phases were collected, added with anhydrous sodium sulphate and evaporated to dryness. Samples dissolved in 50 µl of CHCl_3_ were derivatized as corresponding trimethylsilyl ether by adding 150 µl of bis(trimethylsilyl)trifluoroacetamide and heated for 1 h at 60°C. The samples were evaporated to dryness under a gentle stream of N_2_ and then redissolved in 1 ml of CHCl_3_. GC-FID (Trace 2000, Thermo-Fisher) analyses were performed with a fused-silica capillary column (30 m length × 0.25 mm i.d. × 0.25 µm film thickness, Agilent J&W). The oven was temperature-programmed from 180°C (3 min) to 280°C (5 min) at a rate of 10°C/min and to 300°C (10 min) at a rate of 10°C/min. The temperatures of the injector and detector were 250 and 280°C, respectively. The sample volume was 1 μl with a split injection (1/20). Preliminary peak identification was carried out by comparison of retention times with known standards and external calibration was carried out.

### Statistical Analysis

Data were checked for normality and homoscedasticity; afterwards, they were analyzed by one-way analysis of variance (ANOVA) or T-Student test. Mean separations were performed using the least significant difference (LSD) test at P = 0.05. Analyses were conducted in R environment (R Core Team, 2017).

## Results

### Cyto-Histological Observations

**Figure 1:**
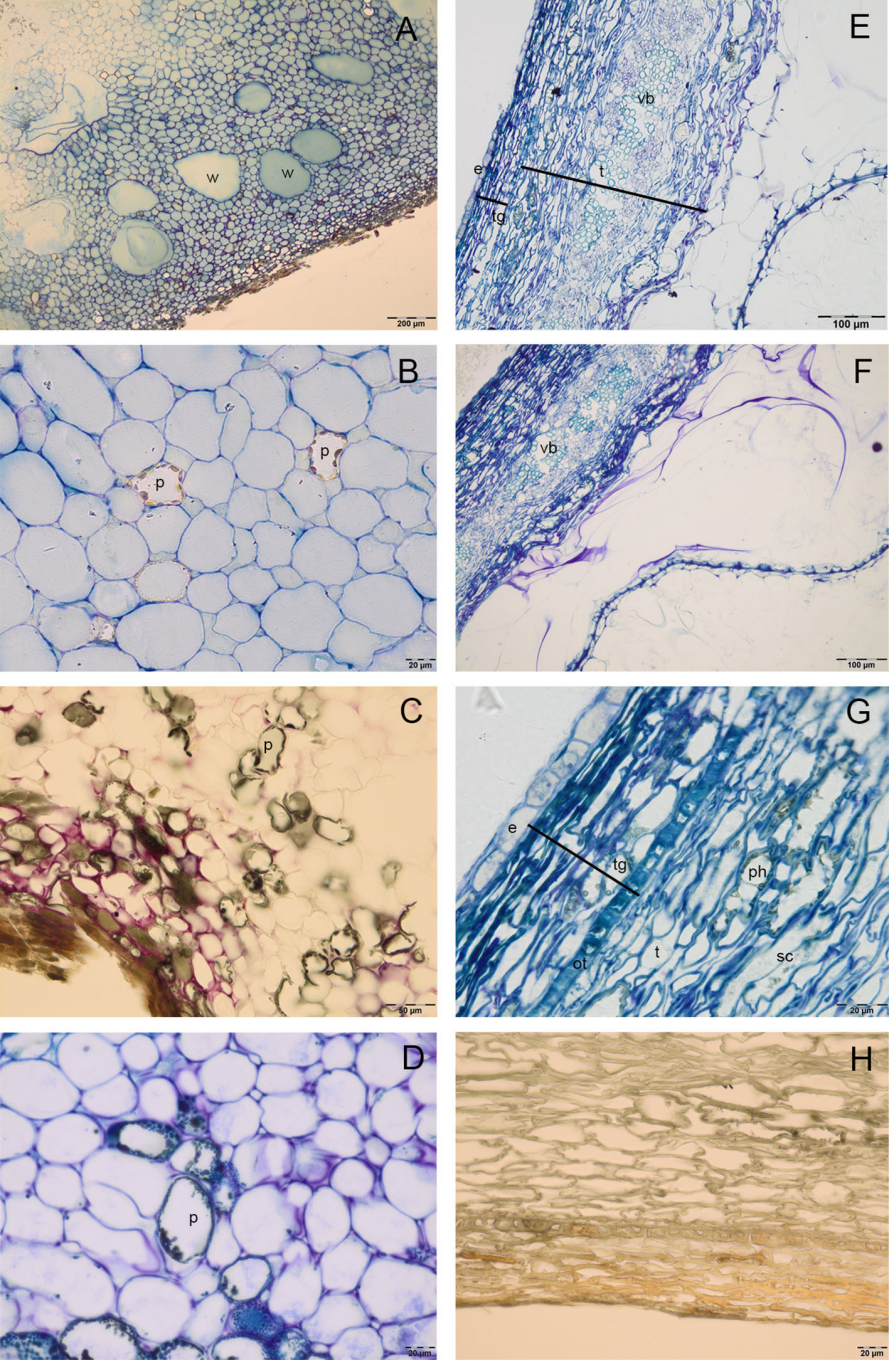
Semithin sections of cocoa fruit and seed. **(A**–**D)** Fleshy mesocarp of pod, stained with toluidine blue **(A**, **B)**, vanillin **(C)** and toluidine blue O **(D)**, in which large cells rich in water (w) and polyphenolic cells (p) are detected. **(E**–**F)** Big tubular cells of endocarp with cell walls rich in pectins, which appeared coloured in magenta after toluidine blue O **(E)** and PAS staining **(F)**; seed tegument constituted by testa (t) and tegmen (tg, in the mesophyll of testa it is possible to distinguish numerous vascular bundles (vb). **(G)** Single layered endosperm (e) adheres to seed tegument, in the tegmen we can identify an outer epidermis (ot), composed by sclerified cells (sc) and more layers of crashed cells; polyphenolic substances (ph) are evident in the region of sclerified cells of testa. **(H)** Tegument stained with vanillin: no epicatechin or catechin presence was detected.

Cocoa pod was characterized by a thin epicarp, a fleshy mesocarp and the endocarp. In the mesocarp, large cells rich in water were alternated with smaller polyphenolic cells (**Figures 1A**–**D**), in which vesicles of polyphenolic substances were localized in the periphery of cells; numerous gaps were also observed. In the endocarp, big tubular cells with large intercellular spaces were detected; the periodic acid–Schiff (PAS) and toluidine blue O staining showed the presence of pectins (coloured in magenta) in the wall of these cells (**Figures 1E**, **F**). Endocarp cells formed, in ripe seeds, a mucilaginous pulp that adhered tightly to seed coat (**Figures 1E**, **F**). In the seed coat, testa was constituted by an exotesta, a single layer of elongated cells, a mesophyll, in which there were numerous vascular bundles, and lignified stellate cells (**Figures 1F**, **G**). In tegmen it was possible to distinguish the outer epidermis, composed by one layer of sclereid cells with a thickened wall, many layers of crushed cells and the inner single-layered epidermis. In T polyphenolic substances were evident above all in the region of lignified stellate cells of testa (**Figure 1G**). The secondary endosperm was constituted by a single layer of cells, with lightly thickened outer walls (**Figure 1G**); it adhered to the seed coat and to numerous folding of the cotyledon tissue. In the T (**Figure 1H**) or mesocarp (**Figure 1C**), the vanillin staining did not reveal the presence of catechin, epicatechin or proanthocyanidins.

**Figure 2:**
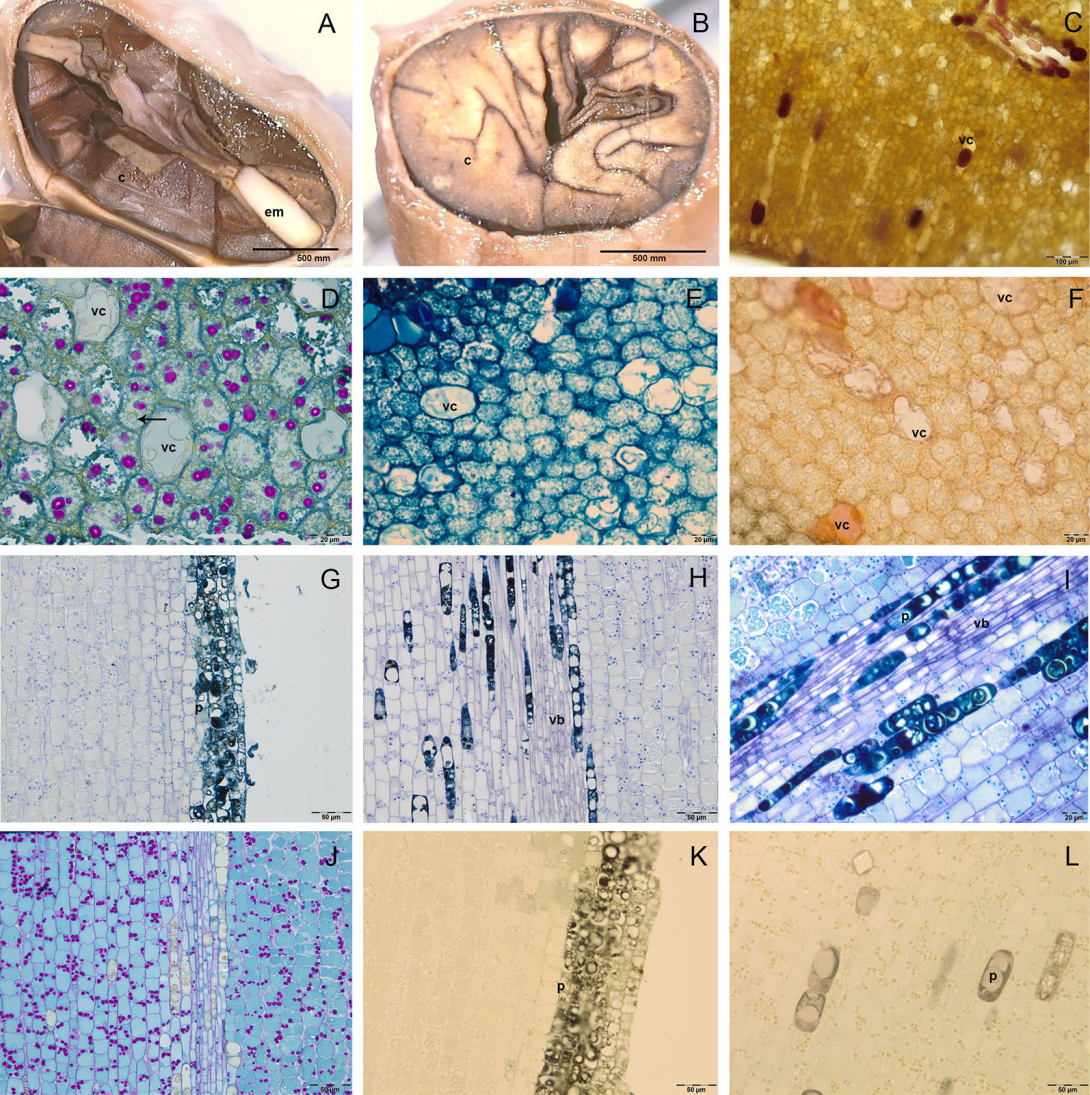
Semithin **(D**–**L)** and fresh **(A**–**C)** sections of cocoa fruit and seed. **(A**–**B)** Fresh section of cocoa seeds: embryo axis (em) and brown cotyledons (c) are evident; cotyledons are foliaceous near the embryo axis and then become fleshy and densely folded. **(C)** Fresh section of cotyledons with big vacuolated cells (vc) rich in anthocyanins. **(D**–**F)** Semi-thin sections of cotyledons stained with periodic acid–Schiff (PAS) **(D)**, toluidine blue O **(E)**, vanillin **(F)**; in cotyledons mesophyll, big vacuolated cells (vc) appeared rich in epicatechin after vanillin staining **(F)**, whilst small cells showed lipid drops (black arrows) and starch grains coloured in magenta after PAS staining. **(G**–**L)** Semi-thin sections of embryo axis, stained with toluidine blue **(G**, **H)**, toluidine blue O **(I)**, PAS **(J)**, vanillin **(K**, **L)**; polyphenolic cells (p) are evident in the superficial layers **(G)** and near the vascular bundles (vb) **(H**, **I)**; the presence of starch grains (magenta colour) is evident **(J)**; no catechin or epicatechin were detected **(K**, **L)**.

The C were foliaceous and densely folded near the EA, then became fleshy and darker for the anthocyanins synthesis (**Figures 2A**, **B**); they were formed by a two single-layered epidermis and a mesophyll rich in reserve substances. In the cotyledon mesophyll it was possible to distinguish two kind of cells: big vacuolated cells, containing vesicles of different size, which can be identified as polyphenolic cells; smaller isodiametric cells, rich in cytoplasm (**Figures 2C**–**F**). Fresh sections showed the presence of anthocyanins in some of the big vacuolated cells, which appeared in some cases also rich in phenolic compounds after staining with toluidine blue O (**Figure 2E**). Vanilline staining demonstrated the localization in some of these cells of catechin and epicatechin (**Figures 2C**, **F**). In the small isodiametric cells, PAS staining demonstrated the presence of starch grains (magenta colour) (**Figure 2D**); in the same cells lipid bodies were also detected (**Figure 2D**).

In the embryo axis, constituted by isodiametric tightly stuck cells arranged in parallel lines, the toluidine blue staining sharpened the presence of 5/6 superficial layers of polyphenolic cells (**Figure 2G**), which were observed also near to vascular bundles located in the center of embryo axis (**Figure 2H**). The polyphenolic content of these cells was confirmed also by staining with toluidine blue O (**Figure 2I**). In the same portions, PAS staining demonstrated also the presence of starch grains (**Figure 2J**). No catechin or epicatechin were detected through vanillin staining (**Figures 2K**, **L**).

### Phytochemical Analysis

**Figure 3:**
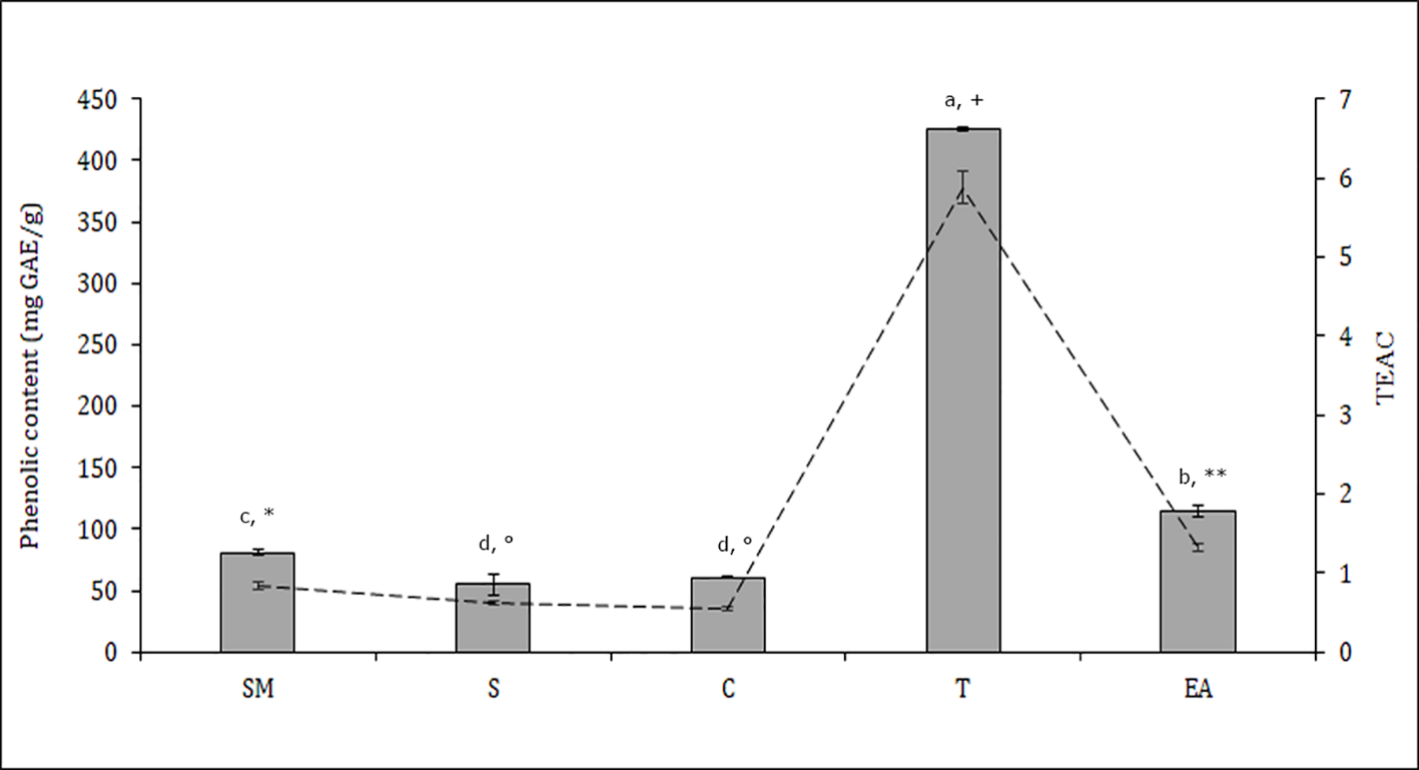
Total phenolic content (bars) and antioxidant activity Trolox equivalent antioxidant capacity (TEAC) (dashed line) in different *Theobroma cacao* seed samples; T, tegument; C, cotyledons; SM, whole seed; S, seed without mucilage; EA, embryo axis (see *Materials and Methods*). The values represent means ± SD of three replicates. Different letters and symbols indicate statistically significant differences according to LSD test (p ≤ 0.05) for phenolic content and TEAC, respectively.

**Figure 4:**
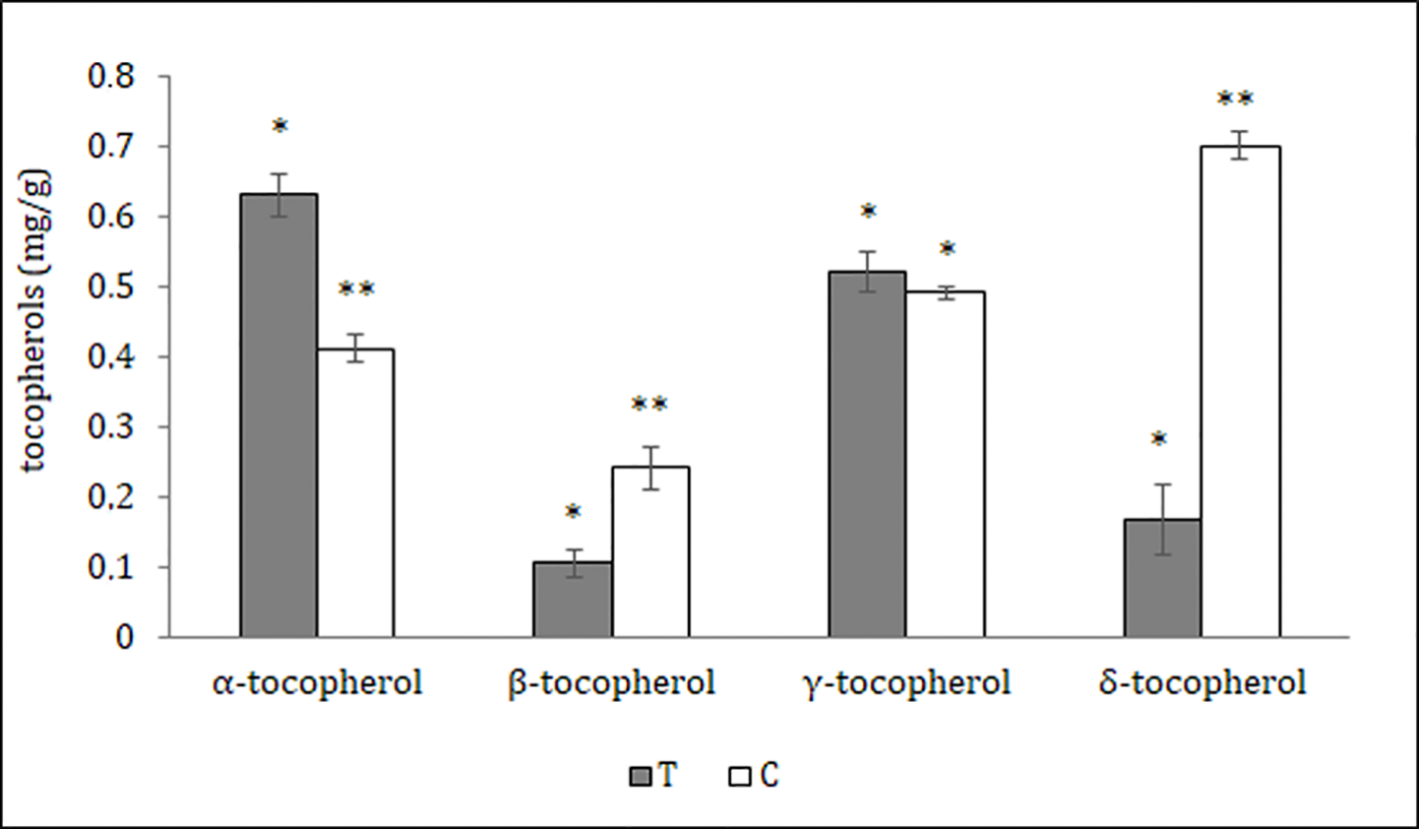
Tocopherols content of different *Theobroma cacao* seed parts; T, tegument; C, cotyledons (see *Materials and Methods*). The results are expressed in milligram/gram sample ± SD of three replicates. Different symbols indicate statistically significant differences according to least significant difference test (p ≤ 0.05).

The distribution of polyphenols, tocopherols, fatty acids and sterols were also evaluated in the different seed parts with the aim to better characterize them and to confirm the histological data. Particularly relevant was the significantly highest abundance of total polyphenols in the T, data confirmed by the results of the antioxidant activity (TEAC value) of the different cocoa extracts (**Figure 3**). The content of polyphenols and TEAC were significantly lower in S (seed without mucilage) than in the SM (seed with mucilage) sample, showing that the mucilage contributes greatly to the content of these phytochemicals and to the antioxidant activity. The individual phenolic content was investigated in the samples T, C, S, SM. As shown in **Table 1**, whole seed samples, S and SM, were qualitatively richer in phenolic compounds than the other isolated seed components. Moreover, in S and SM samples there was a significantly higher abundance of chlorogenic acid, hydroxycinnamic, vanillic and ferulic acid. Caffeic and syringic acids were, instead, more abundant in the T; caffeic acid together with p-cumaric and protocathetic acid were the only components present in all the analyzed samples. Catechin and epicatechin were not detected in the T but present in C, SM and S; particularly epicatechin was the most abundant compound isolated in S, SM and C sample (e.g., 34% of the total phenols in C). Quercitin was not detected in any analyzed samples. The total tocopherols content was analyzed in the single components of seed, T and C samples; it was higher in C with respect to the T (1.84 ± 0.07 *vs.* 1.43 ± 0.04 mg/g). The distribution of the single isomers can be different in these two parts of the seed; this is true for α, β and δ-tocopherol but not for γ-tocopherol (**Figure 4**); indeed β and δ-tocopherol were higher in C whilst the T was richer in α-tocopherol.

**Table 1:** Phenolic compounds content in different *Theobroma cacao* seed components.

Phenolic compound	T	C	SM	S
Chlorogenic acid	n.d.	8.3 ± 0.80 ^f,†^	10.52 ± 0.84 ^f,^^‡^	11.32 ± 0.63 ^d,•^
Ferulic acid	n.d.	12.45 ± 1.26 ^c,^^†^	22.62 ± 1.65 ^c,^^•^	18.51 ± 1.51 ^b,‡^
Caffeic acid	18.7 ± 0.94 ^a,•^	8.92 ± 0.58 ^e,†^	13.45 ± 2.54 ^e,‡^	4.51 ± 0.64 ^g,^^*^
p-Cumaric acid	10.8 ± 1.84 ^b,‡^	7.54 ± ^1.2 g,†^	14.52 ± 1.64 ^d,•^	10.21 ± 2.15 ^e,^^‡^
p-Hydroxycinnamic acid	n.d.	13.07 ± 1.5 ^b,†^	23.41 ± 3.54 ^b,•^	13.71 ± 1.82 ^c,‡^
p-Hydroxybenzoic acid	n.d.	10.41 ± 2.1^d,•^	0.65 ± 0.17 ^k,†^	2.61 ± 0.50 ^i,‡^
Syringic acid	6.21 ± 0.58 ^c,•^	n.d.	4.25 ± 0.08 ^g,‡^	3.43 ± 0.20 ^h,^^†^
Vanillic acid	3.04 ± 0.06 ^d,†^	n.d.	3.65 ± 1.24 ^h,‡^	6.14 ± 0.42 ^f,•^
Protocathetic acid	2.61 ± 0.05 ^d,•^	1.8 ± 0.04 ^†,h^	2.85 ± 0.10 ^i,•^	2.16 ± 0.40 ^j,‡^
Catechin	n.d.	0.61 ± 0.02 ^i,†^	1.21 ± 0.06 ^j,^^•^	0.96 ± 0.08 ^k,^^‡^
Epicatechin	n.d.	34.25 ± 1.34 ^a,•^	32.15 ± 2.54 ^a,†^	33.4 ± 0.95 ^a,^^‡^
Quercetin	n.d.	n.d.	n.d.	n.d.

T, tegument; C, cotyledons; SM, whole seed; S, seed without mucilage (see Materials and Methods). Results are expressed as mean milligram/gram sample ± SD of three replicates. In each column, different letters indicate statistically significant differences according to least significant difference (LSD) test (p ≤ 0.05); in each row, different symbols indicate statistically significant differences according to LSD test (p ≤ 0.05). n.d., not detected.

The phytosterols characterization of seed parts showed a qualitative homogenous distribution with a quantitative predominance of β-sitosterol in all the three seed components (T, C, EA) with respect to the others (**Table 2**). In our samples the concentrations of the single compounds were significantly higher in T in comparison to the other seed parts, except for β-sitosterol and Δ5-avenasterol that were detected in significantly larger amount in the embryo-axis (**Table 2**).

**Table 2:** Sterols content in different *Theobroma cacao* seed components.

Sample	Campesterol	Campestanol	Stigmasterol	β-Sitosterol	Δ5-Avenasterol
T	11.2 ± 1.2 ^a,□^	15.8 ± 0.6 ^a,*^	18.6 ± 2.6 ^a,†^	62.3 ± 3.5 ^b,•^	29.6 ± 0.5 ^b,‡^
C	5.62 ± 0.8 ^b,□^	8.6 ± 1.1 ^c,^^*^	16.2 ± 0.6 ^b,†^	52.3 ± 5.1 ^c,•^	23.6 ± 3.5 ^c,^^‡^
EA	1.36 ± 0.3 ^c,□^	12.1 ± 1.8 ^b,^^†^	8.6 ± 1.7 ^c,^^*^	72.3 ± 6.42 ^a,•^	42.3 ± 1.4 ^a,‡^

T, tegument; C, cotyledons; EA, embryo axis (see Materials and Results are expressed as mean milligram/gram sample ± SD of three replicates. In each column, different letters indicate statistically significant differences according to least significant difference (LSD) test (p ≤ 0.05); in each row, different symbols indicate statistically significant differences according to LSD test (p ≤ 0.05).

The fatty acids profiles showed a homogeneous content of saturated (SFA; palmitic, stearic and arachidic) and unsaturated (UFA, palmitoleic, oleic, linoleic and linolenic) fatty acids among the different seed components (**Table 3**). The oleic acid C18:1 was, in all samples, the most representative unsaturated fatty acid, whilst stearic was the most abundant SFA. The SFA content were always higher than UFA; the ratio S/U (saturated *vs.* unsaturated) was in a range of 1.46–1.67.

**Table 3:** Fatty acids content in different *Theobroma cacao* seed components.

Sample	Palmitic acid C16:0	Palmitoleic acid C16:1	Stearic acid C18:0	Oleic acid C18:1	Linoleic acid C18:2	Linolenic acid C18:3	Arachidic acid C20:0
T	19.62 ± 0.6 ^b,†^	9.52 ± 0.8 ^b,□^	49.56 ± 1.6 ^b,•^	41.92 ± 1.2 ^b,‡^	1.27 ± 0.2 ^a,♦^	0.23 ± 0.05 ^a,♦^	13.03 ± 0.2 ^a,*^
C	22.21 ± 1.2 ^a,†^	5.23 ± 0.7 ^c,□^	43.56 ± 2.1 ^c,‡^	46.95 ± 2.7 ^a,•^	0.36 ± 0.02 ^b,♦^	0.28 ± 0.02 ^a,♦^	11.23 ± 0.4 ^*^^a,^
EA	24.68 ± 0.9 ^a,†^	13.55 ± 1.8 ^a,*^	52.36 ± 1.8 a^,•^	37.9 ± 1.8 ^c,‡^	1.06 ± 0.03 ^a,♦^	0.38 ± 0.08 ^a,♦^	11.23 ± 1.2 ^a,□^

T, tegument; C, cotyledons; EA, embryo axis (see Materials and Methods). Results are expressed as relative percentage ± SD of three replicates. In each column, different letters indicate statistically significant differences according to least significant difference (LSD) test (p ≤ 0.05); in each row, different symbols indicate statistically significant differences according to LSD test (p ≤ 0.05).

## Discussion

Recently, the interest about cocoa has increased due to the demonstrated benefit activity of cocoa extracts; they showed cardioprotective effects but also seemed to reduce inflammations and cholesterol levels (Engler and Engler, 2004; Cooper et al., 2008; Tokede et al., 2011). The composition of these extracts has been investigated during the different phases of seed development, but few information is yet available about their distribution in different fruit and seed components.

Our data outlined that antioxidant molecules, as tocopherols and polyphenols, were differentially distributed in cocoa fruit portions [i.e., pulp or mucilage (endocarp), T and C]. The highest polyphenols diversity was observed in the whole seed (with and without mucilage), and a characteristic polyphenol pattern was observed in different portion of the seed. Caffeic acid and p-cumaric acid are involved in lignin synthesis, so the higher amounts of these phenols detected in T respect to C can be probably related to a high presence of cells with lignified walls. In C phenolic compounds were located in big vacuolated polyphenolic cells; in some of these cells the presence of anthocyanins and epicatechin, was also highlighted vanillin staining confirmed the absence of epicatechin and catechin in the T, indeed, as evident in phytochemical analysis, epicatechin is the predominant one in C and absent in the T. Catechin and epicatechin are known to be the most abundant flavonoid compounds in cocoa powder; they play a role in plant immunity, growth regulation and support nutrient uptake and photosynthesis (Natsume et al., 2002; Jalil and Ismail, 2008; Prakash et al., 2019).

The presence of phenolic compounds in pulp was demonstrated by Endraiyani et al. (2016), which outlined the thermolability of these substances, but our cytological observations did not confirm that: the osmium and toluidine blue staining did not highlight phenols in the pulp tissue, probably because they are dissolved in the big vacuoles. Polyphenolic substances were, instead, observed in periphery cells of mesocarp, where they appeared reorganized in round vesicles, probably due to fixing in glutaraldehyde (Martini et al., 2008). They were also well evident in the embryo axis, where they localized in the external layers and near the vascular tissues. Even if also non-phenolic substances can be responsible for the antioxidant activity, phenolic content could be used as an indicator of antioxidant activity (TEAC), which was the highest in the T. In T, polyphenolic cells were evident in the testa but the histological localization of few polyphenolic cells in our samples did not seem to justify the high presence detected by chemical analysis. Moreover, TEAC was higher in SM than in S: this is probably connected to a higher presence of ferulic acid, caffeic acid, cumaric acid and syringic acid in the seed with mucilage. Other important components of cocoa beans are represented by fatty acids and phytosterols; the content of saturated and unsaturated fatty acids, bound in triglycerides, influence cocoa butter hardness and consequently its commercial value.

Among the analyzed fatty acids, the stearic and oleic acids were the most abundant in all the seed components (T, C and embryo axis). In particular the content of stearic acid is higher in embryos with respect the other seed parts whereas the amount of oleic acid is higher in C. Stearic acid is particularly appreciated, as it did not seem to increase the level of total cholesterol and low-density lipoprotein (LDL)-cholesterol in serum unlike the other saturated acid (Yu et al., 1995). In embryos the C16:0 and C18:0 were the predominant SFA, and this represent an advantage because the yield of ATP molecules during complete oxidation is higher than UFA.

Fatty acids, either free or as part of complex lipids, are important constituent of membranes or other cellular structures but also represent one of the storage substances of cocoa seed probably localized in lipid globules. In cocoa, as observed by Bayés-García et al. (2019), lipid droplets or lipid globules represent almost the 50% (w/w) in fresh cacao beans and serve as important reservoirs of lipids, but also as substrates for multiple cellular processes; in our observations they occupied almost the entire volume of small isodiametric cells of cotyledon mesophyll.

The cocoa seeds were also rich in plant sterols, which had protective effects on the oxidation of lipids as result of synergistic interactions with tocopherols (Caporaso et al., 2018); they also regulate the fluidity and permeability of plant cell membranes, in a similar manner as cholesterol in mammalian. Moreover, these compounds have a beneficial effect on the human health as they seemed to contribute to reduce the level of LDL cholesterol in blood serum (Wollgast and Anklam, 2000; Andújar et al., 2012). In cocoa seeds, we observed a different distribution of the analyzed sterols: β-sitosterol and Δ5-avenasterol were the most abundant, above all in the embryo axis; stigmasterol and campesterol were less present in EA and more abundant in T; campestanol level was again higher in T but lower in C. The specific localization of different kind of sterols was related probably with a peculiar function. Stigmasterol, for instance, is generally not involved in the regulation of membrane characteristics, but in cell proliferation and proton pumping (Hartmann, 1998). Tocopherol also showed a differential distribution in T and C. Content, composition and presence of tocopherols varies widely in different plant tissues, moreover, both plant growth and development affect the levels of tocopherol content and composition, which changes for example during senescence, chloroplast to chromoplast conversion, fruit ripening and seed development (Arrom and Munné-Bosch, 2010; Falk and Munne-Bosh, 2010).

## Conclusions

This study provides for the first time a whole picture of the distribution of many important compounds in cocoa, as until now only the localization of single chemicals in specific seed structures had been investigated (Dangou et al., 2002; Martini et al., 2008; Elwers et al., 2010). New information about cyto-histological characteristics of cocoa seeds were also reported, as the storage role of big vacuolated cells present in C tissue. These cells can accumulate different compounds; anthocyanins and epicatechins were for example detected only in some of them.

## Data Availability Statement

The datasets generated for this study are available on request to the corresponding author.

## Author Contributions

LR conceived the work. MC and LR performed microscopy analyses. CZ performed chemical analyses. MC performed statistical analyses. LR drafted the manuscript and MC and CZ critically revised the draft. All the authors approved the version of the manuscript to be published.

## Conflict of Interest

The authors declare that the research was conducted in the absence of any commercial or financial relationships that could be construed as a potential conflict of interest
